# Altered Sexual Behavior in Dopamine Transporter (DAT) Knockout Male Rats: A Behavioral, Neurochemical and Intracerebral Microdialysis Study

**DOI:** 10.3389/fnbeh.2020.00058

**Published:** 2020-04-20

**Authors:** Fabrizio Sanna, Jessica Bratzu, Maria Pina Serra, Damiana Leo, Marina Quartu, Marianna Boi, Stefano Espinoza, Raul R. Gainetdinov, Maria Rosaria Melis, Antonio Argiolas

**Affiliations:** ^1^Department of Biomedical Sciences, Section of Neuroscience and Clinical Pharmacology, Centre of Excellence for the Neurobiology of Addictions, University of Cagliari, Cagliari, Italy; ^2^Department of Biomedical Sciences, Section of Citomorphology, University of Cagliari, Cagliari, Italy; ^3^Department of Neurosciences, University of Mons, Mons, Belgium; ^4^Department of Neuroscience and Brain Technologies, Fondazione Istituto Italiano di Tecnologia, Genoa, Italy; ^5^Institute of Translational Biomedicine, St. Petersburg State University, St. Petersburg, Russia; ^6^Institute of Neuroscience, National Research Council, Cagliari Section, Cagliari, Italy

**Keywords:** sexual behavior, DAT knockout rats, dopamine, glutamic acid, D-FosB, BDNF/trkB, synaptic proteins, Arc

## Abstract

Central dopamine plays a key role in sexual behavior. Recently, a Dopamine Transporter knockout (DAT KO) rat has been developed, which displays several behavioral dysfunctions that have been related to increased extracellular dopamine levels and altered dopamine turnover secondary to DAT gene silencing. This prompted us to characterize the sexual behavior of these DAT KO rats and their heterozygote (HET) and wild type (WT) counterparts in classical copulatory tests with a sexually receptive female rat and to verify if and how the acquisition of sexual experience changes along five copulatory tests in these rat lines. Extracellular dopamine and glutamic acid concentrations were also measured in the dialysate obtained by intracerebral microdialysis from the nucleus accumbens (Acb) shell of DAT KO, HET and WT rats, which underwent five copulatory tests, when put in the presence of an inaccessible sexually receptive female rat and when copulation was allowed. Markers of neurotropism (BDNF, trkB), neural activation (Δ-FosB), functional (Arc and PSA-NCAM) and structural synaptic plasticity (synaptophysin, syntaxin-3, PSD-95) were also measured in the ventral tegmental area (VTA), Acb (shell and core) and medial prefrontal cortex (mPFC) by Western Blot assays. The results indicate that the sexual behavior of DAT KO vs. HET and WT rats shows peculiar differences, mainly due to a more rapid acquisition of stable sexual activity levels and to higher levels of sexual motivation and activity. These differences occurred with differential changes in dopamine and glutamic acid concentrations in Acb dialysates during sexual behavior, with lower increases of dopamine and glutamic acid in DAT KO vs. WT and HET rats, and a lower expression of the markers investigated, mainly in the mPFC, in DAT KO vs. WT rats. Together these findings confirm a key role of dopamine in sexual behavior and provide evidence that the permanently high levels of dopamine triggered by DAT gene silencing cause alterations in both the frontocortical glutamatergic neurons projecting to the Acb and VTA and in the mesolimbic dopaminergic neurons, leading to specific brain regional changes in trophic support and neuroplastic processes, which may have a role in the sexual behavior differences found among the three rat genotypes.

## Introduction

Brain dopamine is involved in both motivational and consummatory aspects of male sexual behavior. Among brain areas that mediate the sexual roles of dopamine, the most studied are hypothalamic nuclei, as the paraventricular nucleus of the hypothalamus (PVN) and the medial preoptic area, the ventral tegmental area (VTA), the nucleus accumbens (Acb) and the prefrontal cortex (PFC). While the PVN receives the synapses of the incertohypothalamic dopaminergic neurons (originating in the catecholaminergic A3 and A4 groups, see Dahlström and Fuxe, 1964), the VTA contains the cell bodies of mesolimbic and mesocortical dopaminergic neurons, which send their projections to the Acb and PFC (Everitt, 1990; Pfaus and Phillips, 1991; Hull et al., 1995; Melis and Argiolas, 1995, 2011; Pfaus and Everitt, 1995; Argiolas and Melis, 1995, 2005, 2013; Pfaus, 2010; Sanna et al., 2011, 2012; Hull and Dominguez, 2015). Accordingly, alterations/differences in dopamine function in these areas can substantially affect several aspects of sexual behavior. For instance, we have recently reported (Sanna et al., 2014a) significant differences in both motivational and performance aspects of sexual behavior between Roman High- and Low-Avoidance (RHA and RLA) rats, which are two lines of rats psychogenetically selected for their extremely divergent acquisition of the active avoidance response in the shuttle box, and which display opposite biobehavioral traits (Giorgi et al., 2007, 2019). The sexual differences between the two Roman rat lines seem to be related, at least in part, to differences in dopamine function (Sanna et al., 2013, 2014b), in particular in the tone of mesolimbic and mesocortical dopaminergic neurons at the level of the Acb (Sanna et al., 2015b) and of the medial PFC (mPFC; Sanna et al., 2017b). Accordingly, both naïve (i.e., never exposed before to sexual stimuli) and sexually experienced RHA rats (i.e., exposed to five preliminary copulatory tests), which displayed higher dopamine increases in the Acb and mPFC when exposed to a sexually receptive female rat, displayed also higher sexual motivation and better copulatory performances (i.e., higher ejaculation frequency and intromission ratio and shorter post-ejaculatory interval and latencies to mount, intromit and ejaculate) when compared to their RLA counterparts. Sexually naïve and sexually experienced RHA and RLA rats showed also significant differences in the expression of molecules considered as markers of neural activation (i.e., C-Fos and Δ-FosB) and plasticity [Brain-Derived Neurotrophic Factor (BDNF), the tyrosine kinase receptor B (trkB), and Arc] in the VTA, Acb (shell and core) and mPFC after the exposition to, and sexual interaction with, a sexually receptive female rat (Sanna et al., 2019). In particular, RHA rats displayed higher levels of C-Fos, Δ-FosB and Arc after sexual activity than their RLA counterparts and these differences were very evident in naïve animals being reduced, although not completely, in the experienced ones (Sanna et al., 2019).

Worth noting, Roman RHA and RLA rat lines are not the only ones that display different patterns of sexual behavior concomitant to a different monoaminergic (i.e., dopaminergic and/or noradrenergic) tone. This has been found also in high and low novelty exploration responders (bNEHR and bNELR) rat lines (Cummings et al., 2013) and the High- and Low-yawning (HY and LY) rat lines (Eguibar et al., 2016) (for a review on the specific features of sexual behavior of these rat lines see Melis et al., 2019). Together these findings not only confirm the involvement of dopamine in sexual behavior (Pfaus, 2010) but also show that differences in dopamine function at the level of specific brain areas (e.g., VTA, Acb, and mPFC) may be responsible for differences in several aspects of physiological and pathological sexual behavior as well as, more in general, in motivated behavior (Melis et al., 2019).

Recently, a novel strain of knockout rats for the plasma membrane dopamine transporter DAT (DAT KO rats) has been developed by silencing the gene encoding DAT by using zinc finger nuclease technology (Leo et al., 2018b). DAT KO rats (with total DAT gene silencing) develop normally but weigh less than heterozygote (HET; with partial DAT gene silencing) and wild type (WT; with no DAT gene silencing) rats and show pronounced spontaneous locomotor hyperactivity associated with impairments in cognition (i.e., working memory) and sensory-motor gating. These rats display also impulsive/compulsive traits, stereotypies, anhedonia, asocial profile, alterations in facing novelty and in motivation (Adinolfi et al., 2018, 2019; Cinque et al., 2018; Apryatin et al., 2019; Mariano et al., 2019). To date, these behavioral alterations have been mainly related to a dysfunctional striatal dopamine turnover due to the total or partial DAT gene silencing and to alterations in frontostriatal BDNF, trkB and post-synaptic density protein 95 (PSD-95) levels, leading to consider these animals as a new model for the study of pathological hyperdopaminergic conditions ranging from the attention-deficit/hyperactivity disorder (ADHD) to autism and psychosis spectrum disorders (Leo et al., 2018a).

The availability of these new DAT KO rats prompted us to characterize the sexual behavior of these animals and their HET and WT counterparts in classical copulatory tests with a sexually receptive female rat, to get further insights on the role of dopamine in the male rat sexual behavior. We have first characterized the copulatory pattern of DAT KO rats compared to their HET and WT counterparts and verified if and how sexual behavior changes in following (up to five) copulatory tests done at 3-day intervals from each other, with a sexually receptive female rat. After the characterization of their copulatory patterns, these DAT KO, HET, and WT rats were then implanted with an intracerebral microdialysis probe to measure the extracellular concentration of dopamine in the dialysates from the shell of the Acb, a key area involved in the motivational aspects of sexual behavior (Fiorino et al., 1997; Sanna et al., 2015b) and, more in general, in the transposition of the motivational drive in goal-directed behaviors (Goto and Grace, 2005 and references therein), during both the appetitive (motivation) and consummatory (motivation and performance) phases of sexual behavior. Extracellular glutamic acid concentration was also measured in the same dialysate aliquots used for dopamine measurement, due to the key role of this excitatory amino acid in modulating dopamine activity in the Acb (Britt et al., 2012; Quiroz et al., 2016). Finally, since it has been shown: (i) that dopamine neurotransmission is related to the proper expression of products of the immediate early gene as Δ-FosB (Pitchers et al., 2013) and Arc (Fosnaugh et al., 1995; Managò et al., 2016); (ii) that the dopaminergic dysfunction caused by DAT gene silencing may hamper brain maturation and cause long-lasting impairment of cortico-striatal expression of molecules involved in neurotrophic support and synaptic plasticity such as BDNF, trkB and PSD-95, leading to a persistent reduction in neuronal plasticity and subsequent behavioral alterations (Fumagalli et al., 2003; Yao et al., 2004; Efimova et al., 2016; Leo et al., 2018b); and (iii) that RHA rats, which have a higher dopaminergic tone than RLA rats in the Acb and mPFC, also displayed higher levels of C-Fos, Δ-FosB and Arc after sexual activity than their RLA counterparts together with differential changes in BDNF-trkB system in the VTA, mPFC and Acb (Sanna et al., 2019); we measured not only the expression of the neurotrophic molecules BDNF and trkB (Cunha et al., 2010) and of Δ-FosB, a marker of neural activation (Nestler, 2008; Pitchers et al., 2010b), but also of Arc and PSA-NCAM, two markers of functional synaptic plasticity (see Muller et al., 2000; Bonfanti, 2006; Gascon et al., 2007; Bramham et al., 2010; Korb and Finkbeiner, 2011), and of synaptophysin, syntaxin-3 and PSD-95, markers of structural synaptic plasticity (see El-Husseini et al., 2000; Minzer et al., 2004; Yoon et al., 2007), respectively, by means of Western Blot assays in* ex vivo* tissues of the VTA, mPFC and Acb shell and core of DAT KO, HET and WT rats that underwent intracerebral microdialysis. We hypothesized that: (i) DAT KO, HET, and WT rats should display behavioral differences in several aspects of sexual behavior (e.g., acquisition of sexual experience, motivation, performance) with DAT KO rats displaying a more rapid acquisition of sexual experience (i.e., a stable level of sexual activity) and higher levels of sexual motivation and performance than HET and WT rats, in particular, shorter latencies to mount, intromit and ejaculate, and a higher intromission ratio and ejaculatory frequency; and (ii) these differences should occur concomitantly with differences in the activity of dopamine and/or glutamic acid neurotransmission at the level of the Acb shell, in particular, a higher dopamine and/or glutamic acid activity in DAT KO rats compared to their HET and WT counterparts, and in the expression of one or more markers of neurotrophism, neural activation, functional and structural synaptic plasticity in limbic brain areas relevant for sexual behavior as the VTA, the mPFC and/or the Acb [e.g., DAT KO should be expected to display greater differences than HET compared to WT rats and, in particular, higher levels of Δ-FosB and Arc (Sanna et al., 2019) but lower levels of BDNF, trkB, and PSD-95 (Leo et al., 2018b)].

## Materials and Methods

### Animals

The DAT KO rat line was created in the outbred Wistar Han background at SAGE Labs. The procedures used for the construction, validation, selection, and breeding of the colony have been described in detail elsewhere (Leo et al., 2018b). Male DAT knockout (DAT KO; *N* = 8), heterozygote (HET; *N* = 10) and wild type (WT; *N* = 8) rats (weighing 250–300 g at the beginning of the experiments) were from the colony established at the Italian Institute of Technology, Genoa, Italy. Genotyping was performed by PCR followed by enzymatic digestion with BtsI MutI (New England Biolabs, Milan, Italy). Primers used for PCR amplification were the following: Slc6a3 Cel-1 F 5′-TCCTGGTCAAGGAGCAGAAC-3′, Slc6a3 Cel-1 R 5′-CACAGGTAGGGAAACCTCCA-3′ (Leo et al., 2018b).

Ovariectomized stimulus female rats (*N* = 30, weighing 250–300 g at the beginning of the experiments) used in all the experiments, were obtained from Envigo (San Pietro al Natisone, Italy). Animals were kept 2–4 per cage (38 cm × 60 cm × 20 cm) and were acclimated to the housing facilities of the Department of Biomedical Sciences of the University of Cagliari for at least 10 days before the beginning of the experiments under controlled environmental conditions (24°C, 60% humidity, reversed 12 h light/dark cycle, with lights off from 08:00 to 20:00 h) and with water and standard laboratory food *ad libitum*. To limit the stress due to manipulation during the experiments, each animal was daily handled for approximately 1–2 min throughout the habituation period; also, contact with the animal house maintenance personnel was limited to a single attendant and bedding in the home cages was never changed either the day before or on the day of the experiment. The experiments were performed between 10:00–18:00 h according to the guidelines of the European Communities Directive of September 22, 2010 (2010/63/EU) and the Italian Legislation (D.L. March 4, 2014, n. 26), and approved by the Ethical Committee for Animal Experimentation of the University of Cagliari.

### Experimental Groups

Male DAT KO, HET, and WT rats were used in classical 60 min copulatory tests with an ovariectomized sexually receptive female rat. Oestrus was induced by subcutaneous injections of oestradiol benzoate (200 μg/rat in peanut oil) and progesterone (0.5 mg/rat in peanut oil), 48 and 6 h before the behavioral tests, respectively, and ascertained by May-Grunwald-Giemsa coloration and microscopical examination of vaginal smears 1 h before the experiments (Contini et al., 2018). All DAT KO, HET and WT rats underwent five consecutive copulatory tests at 3 days intervals from each other with an always new sexually receptive female rat (Sanna et al., 2014a,b, 2015a,b, 2017b, 2019). Two days after the last copulatory tests, DAT KO, HET and WT rats underwent stereotaxic surgery for the implantation of the microdialysis probe in the Acb shell.

### Sexual Behavior

The following sexual responses were recorded during the first series of copulatory activity (e.g., from the first mount/intromission to the first intromission after the first ejaculation) of the five preliminary copulatory tests and the microdialysis experiment, by an observer who was not aware of the rat line used: mount and intromission latency (ML and IL, timed from the moment in which the receptive female rat is directly accessible to the male until the first mount and/or the first intromission, respectively); mount and intromission frequency (MF and IF, the number of mounts and intromissions in the first series of copulatory activity, respectively); ejaculation latency (EL, timed from the first intromission in the first series until ejaculation) and post-ejaculatory interval (PEI, timed from the first ejaculation until the next intromission). Intromission ratio (IR, the number of intromissions in the first series divided by the sum of the number of mounts and intromissions in the same series) and the inter-intromission interval (III, the ratio between the ejaculation latency of the first series and the number of intromissions in that series) were also calculated. In addition to the above parameters, the total number of mounts (TMF), of intromissions (TIF) and of ejaculations (EF) in the whole 60 min period of copulation, the total copulatory rate (TCR), calculated by dividing the sum of all the activity periods of the male with the female rat (an activity period was defined from the first mount/intromission in a series of copulatory activity until ejaculation in that series) by the sum of all mounts and intromissions of the whole test and the total intromission ratio (TIR) calculated as the TIF divided by the sum of TIF and TMF were also calculated. Moreover, the number of noncontact penile erections (NCPEs), counted during the 30 min period in which the receptive female rat was inaccessible to the male during the microdialysis experiment (see below), was also recorded (Sachs and Barfield, 1976; Melis et al., 2003; Sanna et al., 2014a,b, 2015a,b, 2017b, 2019; Le Moëne and Ågmo, 2019). Finally, since substantial differences in genital self-grooming, a centrally-mediated, self-directed highly stereotyped behavior that in the context of copulation may indicate self-cleaning and/or self-stimulation (Sachs et al., 1988; Berridge et al., 2005), were observed among the three rat lines during the five copulatory tests, the percent of mounts, intromissions, and ejaculations followed by genital grooming as well as the frequency and duration of genital grooming episodes after mounts, intromissions and ejaculation were recorded for each animal in the first series of copulatory activity during the microdialysis experiment (Sachs et al., 1988).

### Microdialysis in the Acb Shell During Sexual Behavior

The day before the microdialysis experiment, DAT KO, HET and WT rats were stereotaxically implanted (Stoelting Co., Wood Dale, IL, USA), under isoflurane anesthesia (1.5–2%; Harvard Apparatus, Holliston, MA, USA), with a microdialysis probe with a U-shaped dialysis membrane (approximately 2 mm of free surface for dialysis), prepared as previously described (Melis et al., 2003), and aimed unilaterally at the Acb shell (coordinates: 2.0 mm anterior and 0.8 mm lateral to bregma, and 8.0 mm ventral to dura; Paxinos and Watson, 2004). On the day of the experiment, during the dark phase of the cycle, the rats were transferred to a mating cage (45 cm × 30 cm × 24 cm) located in a soundproof room lit by a dim red light. The mating cage contained another small Plexiglas cage (15 cm × 15 cm × 15 cm) with 25 holes (Ø 2 mm) in each vertical wall to allow for visual, olfactory and acoustic communication (Sanna et al., 2015b, 2017b). After a 2 h habituation period, the microdialysis probe was connected *via* polyethylene tubing to a CMA/100 microinfusion pump (Harvard Apparatus, Holliston, MA, USA) and perfused with Ringer’s solution, containing 147 mM NaCl, 3 mM KCl and 1.2 mM CaCl_2_, pH 6.5, at a constant flow rate of 2.5 μl/min. After a 2 h equilibration period, four aliquots of 37.5 μl of Acb dialysates were collected every 15 min in polyethylene tubes kept on ice for the measurement of dopamine and glutamic acid concentrations, as described below. A sexually receptive female rat was then introduced into the small cage located inside the mating cage for 30 min, during which two more dialysate aliquots were collected and NCPEs counted (NCPEs are pheromone-mediated penile erections that male rats show in the presence of an inaccessible sexually receptive female rat that they can see, hear, smell but not touch, and are considered an index of sexual arousal; Sachs et al., 1994; Melis et al., 2003; Sanna et al., 2009). Thirty minutes after the introduction of the female rat, the small cage was removed from the mating cage and sexual interaction/copulation allowed for 60 min, during which four more dialysate aliquots were collected and sexual parameters recorded (see above). After 60 min of copulation, the female rat was removed from the mating cage and one additional dialysate aliquot collected (Pfaus and Everitt, 1995; Melis et al., 2003; Sanna et al., 2015b, 2017b).

### Determination of Dopamine Concentration in Dialysates From the Acb Shell

Dopamine concentration in the dialysate from the Acb shell was measured by high-pressure liquid chromatography (HPLC) on a 7.5 cm × 3.0 mm i.d., Supelcosil C18, 3 μm particle size column (Supelco, Supelchem, Milan, Italy) coupled to electrochemical detection (Coulochem II, ESA, Cambridge, MA, USA) using a 4011 dual cell, as already described (Sanna et al., 2015b, 2017b). Detection was performed in reduction mode with potentials set to +350 and –180 mV. The mobile phase was 0.06 M citrate/acetate pH 4.2, containing methanol 20% v/v, 0.1 mM EDTA, 1 μM triethylamine and 0.03 mM sodium dodecyl sulfate at a flow rate of 0.6 ml/min. The sensitivity of the assay was 0.125 pg.

### Determination of Glutamic Acid Concentration in Dialysates From the Acb Shell

The glutamic acid concentration in the dialysate from the Acb shell was measured in the same dialysate aliquots used for the measurement of dopamine as previously described (Succu et al., 2011; Bratzu et al., 2019). Briefly, glutamic acid concentration was measured in 5 μl aliquots of dialysate added to 5 μl of HClO_4_ 100 mM after pre-column derivatization with orto-phtalaldialdehyde and 2-mercaptoethanol by HPLC. The chromatograph was equipped with an automatic injector, a 15 × 0.4 cm Supelco C18 column, 5 μm particle size, and coupled to fluorescence detection (excitation wavelength: 318 nm; emission wavelength: 452 nm; SFM 25 spectrofluorimeter, Kontron, Milan, Italy). The mobile phase was phosphate buffer 0.1 M, pH 6.2 containing methanol 30% v/v and the flow rate 1 ml/min. The column temperature was maintained at 35°C. The sensitivity of the assay was 10 nM.

### Western Blot

After sacrifice by guillotine, rat brains were rapidly dissected and cooled in dry ice for 15 s, placed in a brain matrix and cut in 2 mm thick coronal slices using the stereotaxic coordinates of the rat brain atlas of Paxinos and Watson (2004) as a reference. Unilateral punches of the Acb shell and core (diameter 1.5 mm), contralateral to the side implanted with the microdialysis probe, and bilateral paramedian punches of the mPFC (diameter 2.5 mm) and the VTA (diameter 3 mm) were collected as previously described (Sanna et al., 2019). For each rat, tissue punches were rapidly frozen at −80°C and homogenized in distilled water containing 2% sodium dodecyl sulfate (SDS; 300 μl/100 mg of tissue) and a cocktail of protease inhibitors (cOmplete^TM^, Mini Protease Inhibitor Cocktail Tablets, Cat# 11697498001, Roche, Basel, Switzerland).

Protein concentrations were determined using the Lowry method (Lowry et al., 1951) with bovine serum albumin as the standard. Proteins, 40 μg for each tissue homogenate, diluted 3:1 in 4× loading buffer (NuPAGE LDS Sample Buffer 4×, Novex ThermoFisher Scientific, Waltham, MA, USA), were heated to 95°C for 7 min and separated by SDS-polyacrylamide gel electrophoresis (SDS-PAGE) using precast polyacrylamide gradient gel (NuPAGE 4–12% Bis-Tris Gel Midi, Novex, ThermoFisher Scientific, Waltham, MA, USA) in the XCell4 Sure-Lock Midi-Cell chamber (ThermoFisher Scientific, Waltham, MA, USA). Internal molecular weight (MW) standards (Precision Plus Protein WesternC Standards, Bio-Rad, Hercules, CA, USA) were run in parallel. Two gels at a time were run for Coomassie staining and immunoblotting, respectively. Proteins for immunoblotting were electrophoretically transferred on a polyvinylidene fluoride membrane (Amersham Hybond-P, GE Healthcare, Little Chalfont, UK) using the Criterion Blotter (Bio-Rad). Blots were blocked by immersion in 20 mM Tris base and 137 mM sodium chloride (TBS), containing 0.1% Tween 20 (TBS/T) and 5% milk powder, for 60 min, at room temperature. The primary antibodies were rabbit polyclonal antibodies against BDNF (Cat# N-20 sc-546, Santa Cruz Biotechnology, Dallas, TX, USA), and trkB [Cat# (794) sc-12, Santa Cruz Biotechnology, Dallas, TX, USA], both diluted 1:1,000, and syntaxin-3 (Cat#ab133750, AbCam, Cambridge, UK), diluted 1:500; rabbit monoclonal antibodies against Δ-FosB (Cat#14695; Cell Signalling Biotechnology, Netherlands) and synaptophysin (Cat#5461; Cell Signalling Biotechnology), both diluted 1:1000; and mouse monoclonal antibodies against PSA-NCAM (Cat# MAB5324, RRID: AB_95211, Merck Millipore, Darmstadt, Germany), PSD-95 (Cat# MAB1596; Merck Millipore) both diluted 1:1,000, and Arc (Cat# sc-17839, RRID: AB_626696; Santa Cruz Biotechnology, Santa Cruz, CA, USA), diluted 1:300 in TBS/T containing 5% milk powder and 0.02% sodium azide. Incubations with primary antiserum were carried out for one night at 4°C. After rinsing in TBS/T, blots were incubated at room temperature, for 60 min, with peroxidase-conjugated goat anti-rabbit serum (Cat#9169, Sigma Aldrich, St. Louis, MO, USA), diluted 1:10,000, and anti-mouse serum (AP124P, Merck Millipore), diluted 1:5,000 in TBS/T. Controls for equal-loading of the wells were obtained by immunostaining the membranes, as above, using a mouse monoclonal antibody against glyceraldehyde-3-phosphate dehydrogenase (GAPDH; MAB374, Merck Millipore), diluted 1:1,000, as the primary antiserum, and a peroxidase-conjugated goat anti-mouse serum (AP124P, Merck Millipore), diluted 1:5,000, as the secondary antiserum. To control for non-specific staining, blots were stripped and incubated with the relevant secondary antiserum. To check for antibody specificity and cross-reactivity, the anti-BDNF antibody was challenged with 200 ng of rhBDNF (Cat# B-257, Alomone Labs, Jerusalem, Israel), while the anti-PSA-NCAM antibody was preabsorbed with 500 ng of the alfa-2–8-linked sialic polymer colominic acid (Cat# sc-239576, Santa Cruz Biotechnology, USA). After rinsing in TBS/T, protein bands were developed using the Clarity Max ECL Substrate (Cat# 1705062, Bio-Rad), according to the protocol provided by the manufacturer, and visualized using the ImageQuant LAS-4000 (GE Healthcare). Approximate MW and relative optical density (O.D.) of the labeled protein bands were evaluated by an examiner who was not aware of the rat line from which the tissue analyzed was obtained. The ratio of the intensity of the BDNF-, trkB-, PSA-NCAM-, Arc-, Syntaxin-3-, Synaptophysin-, PSD-95- and Δ-FosB-positive bands, to the intensity of the GAPDH-positive ones was used to compare the relative expression levels of these proteins in the DAT KO, HET, and WT rats. Image Studio Lite Software (RRID: SCR_014211, Li-Cor^1^) was used to quantify the O.D. of each sample.

### Histology

**Figure 1:**
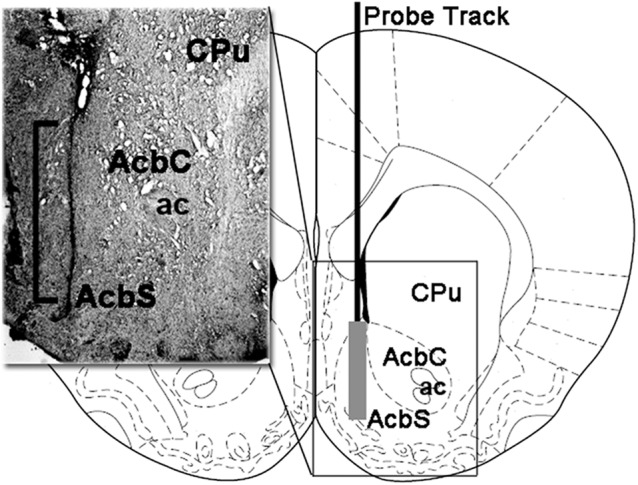
Schematic representation of a coronal section of the rat brain showing the track of the microdialysis probe in the Acb shell (Paxinos and Watson, 2004). Insert: the square bracket in the micro-photograph indicates the portion of the Neutral Red-stained section showing the active part of the dialyzing membrane of the microdialysis probe into the Acb shell. Abbreviations: CPu, caudate-putamen; ac, anterior commissure; AcbS, nucleus accumbens shell; AcbC, nucleus accumbens core.

During the tissue sampling procedure (see above) the hemi-slice containing the track of the microdialysis probe in the Acb shell was collected and immediately stored in 4% aqueous formaldehyde for 12–15 days. Forty micrometre transverse brain sections were then prepared using a freezing microtome, stained with Neutral Red and inspected on a phase-contrast microscope. The position of the tip of the microdialysis probe in the Acb shell was localized by following the tract of the microdialysis probe through a series of brain sections (Figure 1). Only animals with the dialyzing membrane of the microdialysis probe positioned correctly in the Acb shell (eight WT, 10 HET, and eight DAT KO rats) were considered for the statistical evaluation of the results.

### Statistics

**Figure 2:**
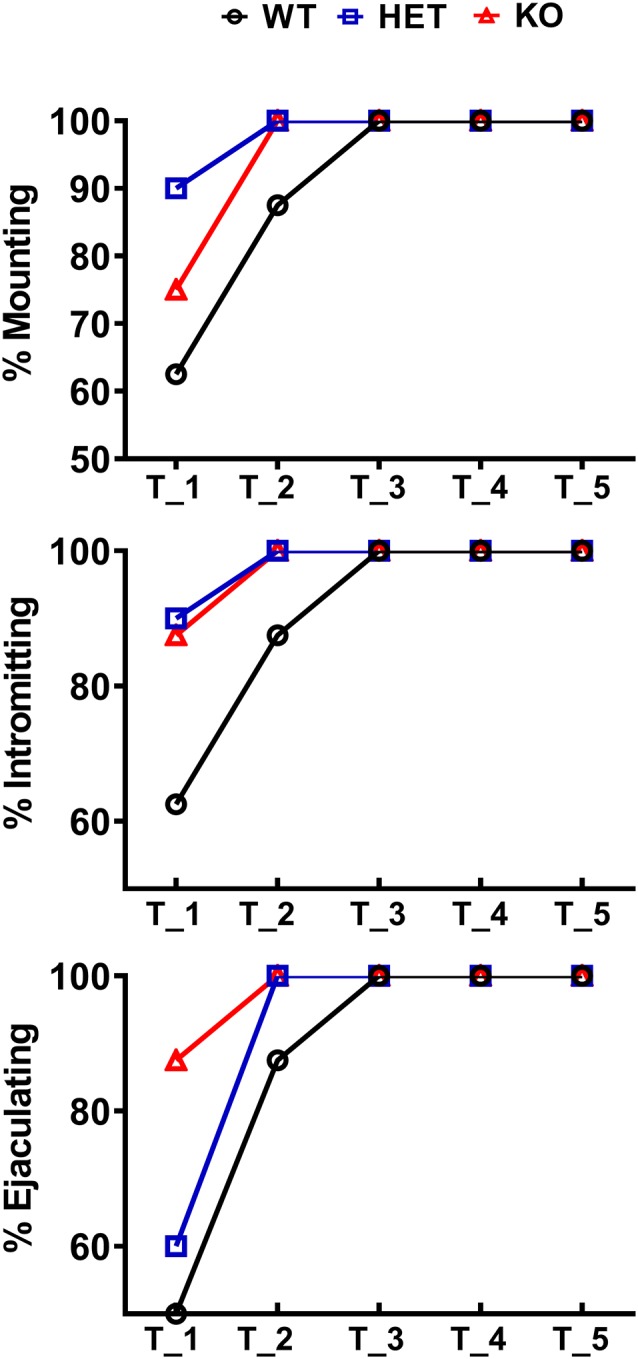
Percentage of male WT, HET and DAT KO rats (8–10 per group) engaged in sexual behavior in copulatory tests 1–5. During each test, male rats were put together with a sexually receptive female rat for 60 min as described in the “Materials and Methods” section. The percent of rats showing mounts, intromissions and that reached ejaculation is reported (Chi-square test, not significant).

**Figure 3:**
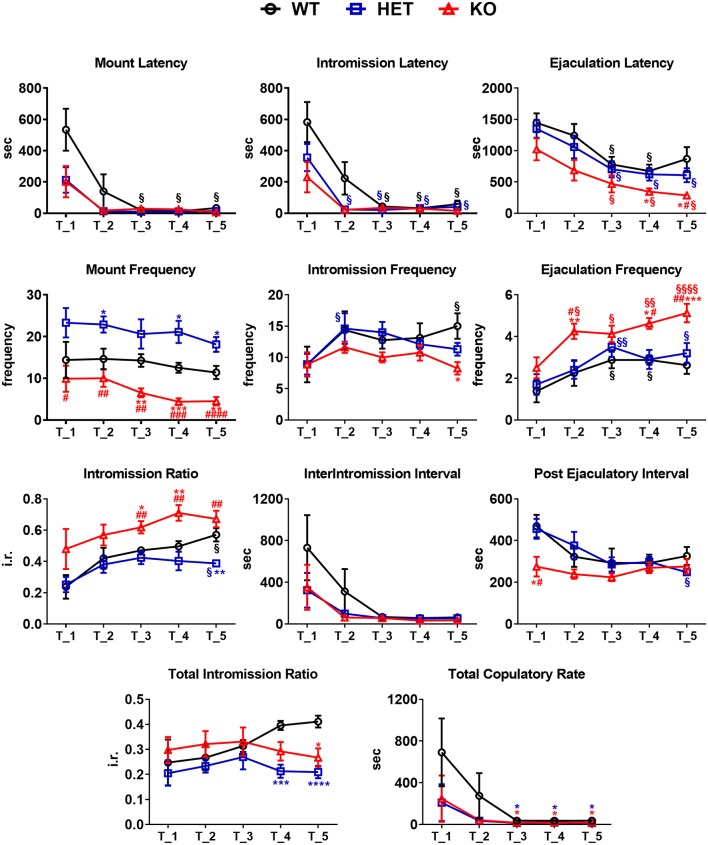
Sexual behavior of male WT, HET and DAT KO rats in the first series of the copulatory test 1, 2, 3, 4 and 5. Male rats were put together with a sexually receptive female rat and observed in order to measure copulatory parameters as described in the “Materials and Methods” section. Copulatory parameters were measured directly or calculated as described in the “Material andMethods” section. Values are means ± SEM of 8/10 rats per group. *P < 0.05, **P < 0.01, ***P < 0.001, ****P < 0.0001 with respect to WT; ^#^*P* < 0.05, ^##^*P* < 0.01, ^###^*P* < 0.001, ^####^*P* < 0.0001, DAT KO with respect to HET; ^§^*P* < 0.05, ^§§^*P* < 0.01, ^§§§§^*P* < 0.0001, with respect to T1 (two-way ANOVA for repeated measures followed by Tukey’s or Bonferroni’s pairwise comparisons).

**Figure 4:**
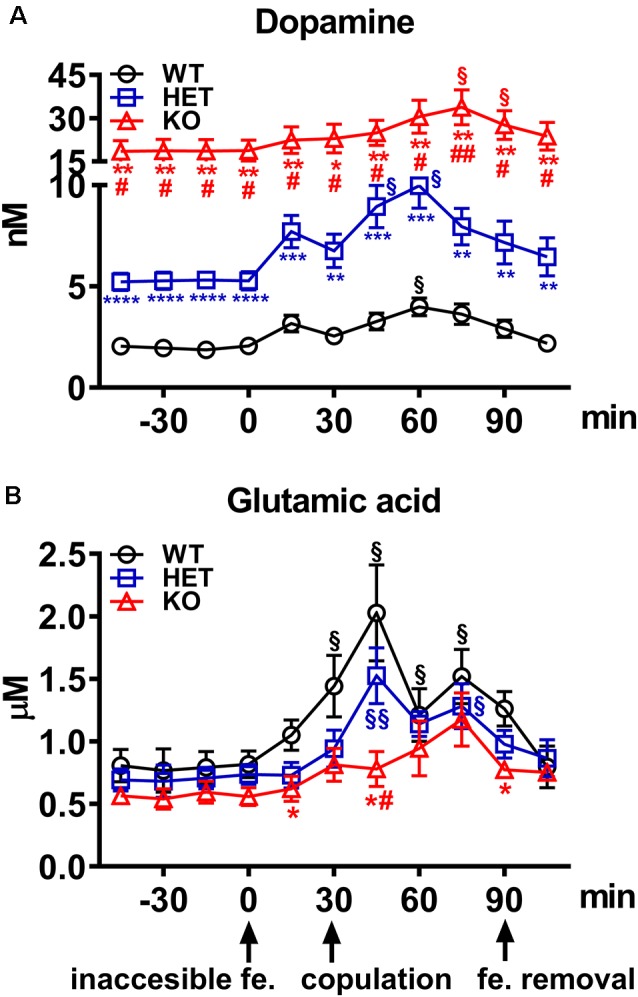
**(A)** Extracellular dopamine and **(B)** glutamic acid concentrations in the Acb shell dialysates obtained from WT, HET and DAT KO rats during sexual activity. Rats from each line, which underwent five copulatory tests with a sexually receptive female rat in the 3 weeks preceding the experiment, stereotaxically implanted with a microdialysis probe aimed at the Acb shell, were placed individually into the mating cage and perfused with the dialysis buffer as described in the “Materials and Methods” section. An inaccessible receptive female rat was then placed inside the small cage of the mating cage (time = 0). After 30 min, copulation was allowed by removing the small cage for 60 min, after which the female rat was removed from the mating cage. During the experiment, NCPEs were counted and copulatory parameters measured, and dialysate aliquots collected every 15 min and analyzed for dopamine and glutamic acid as described in the “Materials and Methods” section. Values are means ± SEM of the values obtained by 8/10 rats per group. ^§^*P* < 0.05, ^§§^*P* < 0.01, with respect to basal values (no female rat); *P < 0.05, **P < 0.01, ***P < 0.001, ****P < 0.0001 with respect to WT; ^#^*P* < 0.05, ^##^*P* < 0.01, DAT KO with respect to HET rats (two-way ANOVA for repeated measures followed by Tukey’s or Bonferroni’s pairwise comparisons).

**Figure 5:**
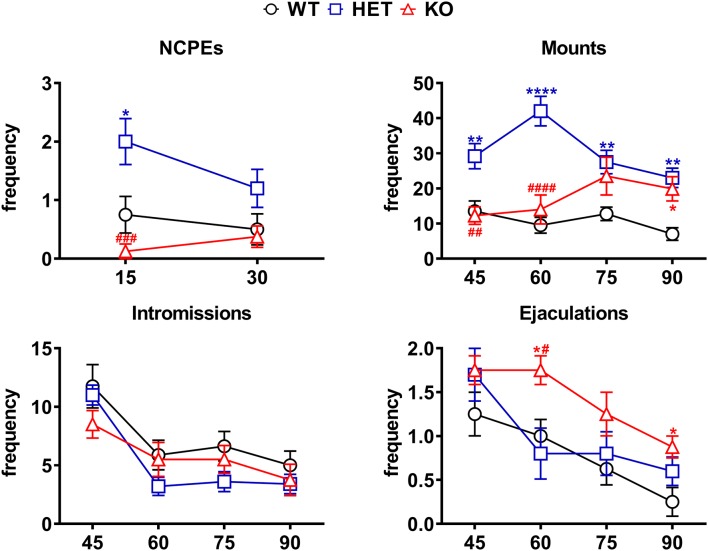
Differences in the number of NCPEs, mounts, intromissions, and ejaculations recorded from WT, HET and DAT KO rats during the microdialysis experiments reported in Figure 4. The experimental conditions were identical to those described in the legend of Figure 4. Values are means ± SEM of the values obtained by 8/10 rats per group. *P < 0.05, **P < 0.01, ****P < 0.0001 with respect to WT; ^#^*P* < 0.05, ^##^*P* < 0.01, ^###^*P* < 0.001, ^####^*P* < 0.0001, DAT KO with respect to HET rats (two-way ANOVA for repeated measures followed by Tukey’s or Bonferroni’s pairwise comparisons).

**Figure 6:**
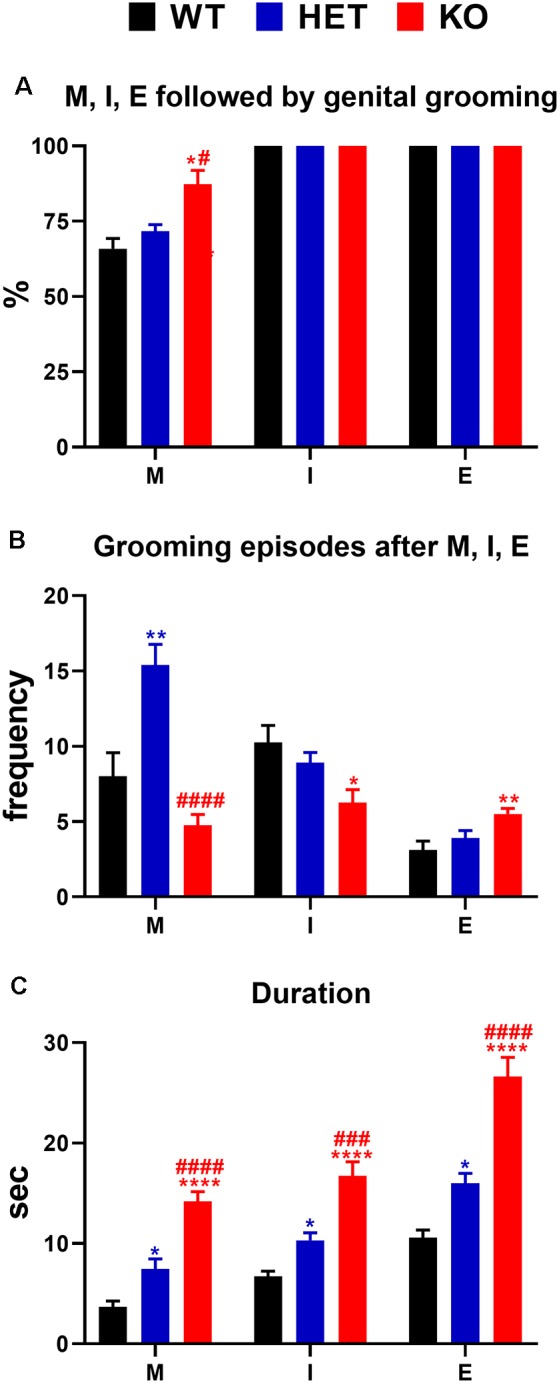
**(A)** Percent of mounts, intromissions, and ejaculations followed by genital self-grooming; **(B)** frequency (i.e., number of episodes of genital self-grooming after mounts, intromissions and ejaculations) and **(C)** duration (s) of genital self-grooming episodes in WT, HET and DAT KO rats after mounts, intromissions and ejaculations in the first series of sexual activity during the microdialysis experiment reported in Figure 4. The experimental conditions were identical to those described in the legend of Figure 4. Values are means ± SEM of the values obtained by 8/10 rats per group. *P < 0.05, **P < 0.01, ****P < 0.0001 vs. WT rats; ^#^*P* < 0.05, ^###^*P* < 0.001, ^####^*P* < 0.0001, DAT KO with respect to HET rats (**A**: Chi-square test; **B,C**: one-way ANOVA followed by Tukey’s pairwise comparisons). M, mounts; I, intromissions; E, ejaculations.

**Figure 7:**
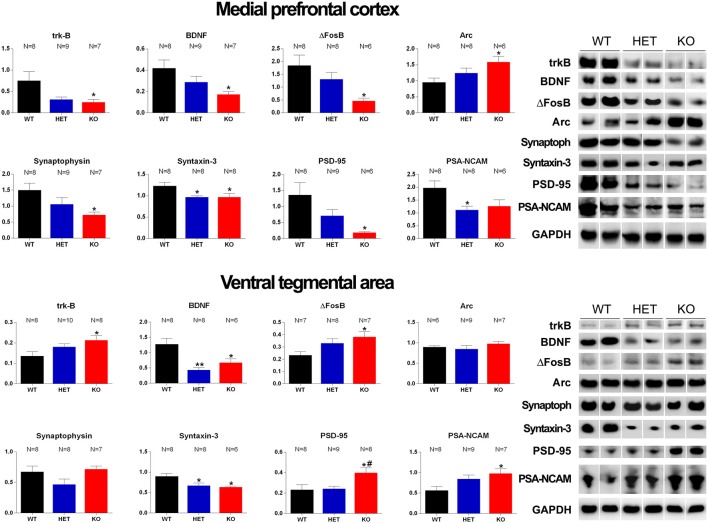
Western Blot analysis of trkB, BDNF, Δ-FosB, Arc, synaptophysin, syntaxin-3, PSD-95 and PSA-NCAM in the mPFC and VTA of WT, HET and DAT KO rats after the microdialysis experiment. Histograms on the left are the densitometric analyses of the marker/GAPDH band gray optical density (O.D.) ratios. Blot lines on the right are representative samples of two animals from each experimental group. Values are means ± SEM of the values obtained by the reported number of rats per group. *P < 0.05, **P < 0.01 with respect to WT rats; ^#^*P* < 0.05 DAT KO with respect to HET rats (one-way ANOVA followed by Tukey’s pairwise comparisons).

**Figure 8:**
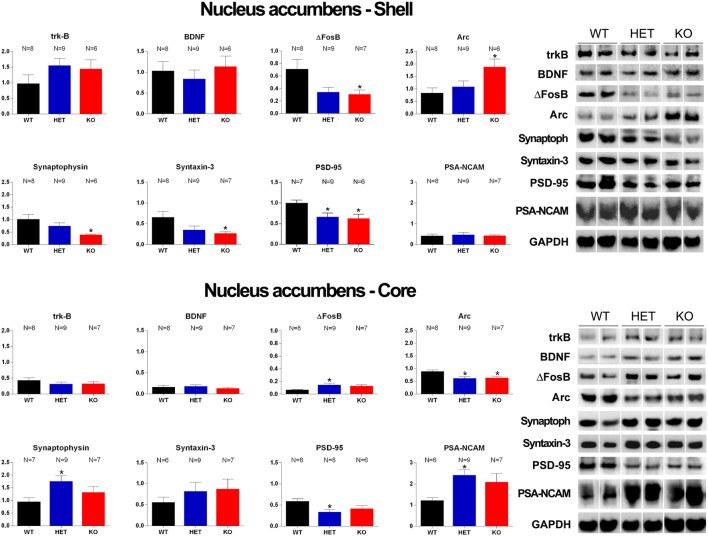
Western Blot analysis of trkB, BDNF, Δ-FosB, Arc, synaptophysin, syntaxin-3, PSD-95 and PSA-NCAM in the Acb shell and Acb core of WT, HET and DAT KO rats after the microdialysis experiment. Histograms on the left are the densitometric analyses of the marker/GAPDH band gray optical density (O.D.) ratios. Blot lines on the right are representative samples of two animals from each experimental group. Values are means ± SEM of the values obtained by the reported number of rats per group. *P < 0.05 with respect to WT rats (one-way ANOVA followed by Tukey’s pairwise comparisons).

Data reported in Figures 2, 6A are presented as a percent of the scored sexual responses and were analyzed using the Chi-square (*χ*^2^) test. All the other data, reported in Figures 3–8, are presented as mean values ± SEM and were analyzed using one- or two-way ANOVAs for repeated measures with the rat line as a between-subjects factor and the time (i.e., copulatory test or dialysate fraction depending on the data set) as a within-subjects factor. When ANOVAs revealed statistically significant main effects and/or interactions, pairwise comparisons were performed by using the Tukey’s multi comparison test or Bonferroni’s corrected multiple t-tests.

Before performing ANOVAs, data sets of each experimental variable were checked for the normal distribution of the values with the Shapiro–Wilk’s test and the homogeneity of variances with the Brown–Forsythe test. When significant differences in the variances of a data set were found, these data were analyzed by means of ANOVAs with the Brown-Forsythe or the Geisser–Greenhouse correction for one- and two-way ANOVAs, respectively.

Animals that did not mount or intromit or ejaculate with the available female rat were assigned the respective full range scores: 900 s for ML and IL when male rats did not mount or intromit within 15 min; 1,800 s for EL when male rats did not ejaculate within 30 min from the first intromission and 600 s for PEI when male rats did not intromit within 10 min after the first ejaculation (Sanna et al., 2014a,b, 2015a,b, 2017b, 2019).

Data from Western blot assays were first analyzed by using the ROUT test (*Q* = 10%) to exclude outliers due to technical problems in collecting/processing the brain tissues (which can lead to erroneous values interfering with the sum-of-the-squares calculation, leading to misleading results) and then analyzed using one-way ANOVAs as described above.

Statistical analyses were all carried out with PRISM, Graph Pad 8 Software (San Diego, USA) with the significance level set at *p* < 0.05.

## Results

### Male DAT KO, HET and WT Rats Display Differences in Sexual Behavior When Exposed to a Sexually Receptive Female Rat

As shown in Figure 2, in the first copulatory test more than 80% of DAT KO and HET rats engaged in copulatory behavior with a sexually receptive female rat, showing mounts and intromissions, but with about 85% of DAT KO rats achieving ejaculation compared to the 60% of HET rats. These values were higher than those of WT rats since only 50–60% of these rats engaged in copulatory behavior with the receptive female rat and achieved ejaculation in the first test. The percent of DAT KO and HET rats showing mounts, intromissions and achieving ejaculation raised to 100% in the second test, as did the WT rats in the third test (Figure 2). Irrespective of the rat line considered, the differences observed in the first and, to a lesser extent, second test among the three rat lines, disappeared in the fourth and fifth tests. However, these differences did not reach statistical significance probably due to the relatively low number of animals used (Chi-square test, Test 1, mounts: *χ*^2^ = 1.918, intromissions: *χ*^2^ = 2.501, ejaculations: *χ*^2^ = 2.693; Test 2, mounts: *χ*^2^ = 2.340, intromissions: *χ*^2^ = 2.340, ejaculations: *χ*^2^ = 2.340, all *Ps* > 0.05). Perhaps more importantly, the above differences occurred together with changes in the copulatory patterns of DAT KO, HET and WT rats (Figure 3) along the five copulatory tests. Accordingly, two-way ANOVA analyses of the parameters recorded in the first series of copulatory activity of the five copulatory tests, revealed significant differences in ML, IL, EL, IF, EF, IR, III, PEI, and TCR along the five tests, and in ML, IL, EL, MF, EF, IR, TIR and PEI among the three rat lines, differences that support a higher level of sexual activity of DAT KO and HET rats compared to WT rats. With the exclusion of the ML and IL, no Line × Test interactions were detected for the other parameters analyzed, a finding that shows that most of the differences observed among the three rat lines during the first copulatory test tended to be conserved along the subsequent ones (Table 1). Moreover, pairwise comparisons (main effect “Test”) also showed that some of the copulatory parameters of the first series of copulatory activity of the first test of DAT KO, HET and WT rats underwent significant changes when compared with those of the first series of copulatory activity of the subsequent tests. Such changes were very evident in all three rat lines when moving from the first to the second and third test, after which no further change was observed in the fourth and fifth test. Infact, when moving from the first to the fifth test, ML, IL, EL and III values decreased and EF and IR values increased in all rat lines, while PEI decreased and IF increased depending on the rat line (see Figure 3 for single points of significance for each rat line). Irrespective of the similar trends found along the five tests among DAT KO, HET and WT rats, pairwise comparisons (main effect “Line”) revealed also that DAT KO rats displayed: (i) a shorter EL and a higher EF, which became both statistically significant in the last two (4th and 5th) tests, and a shorter PEI during the first test when compared to both WT and HET rats; (ii) a significantly lower, and HET rats a significantly higher, MF than WT rats along the five tests; (iii) a significantly higher, and HET rats a significantly lower IR, which became both statistically significant in the last test(s) (tests 3–5), than that of WT rats (see Figure 3 for pairwise single points of significance between rat lines).

**Table 1:** F values and significance levels from two-way ANOVAs for repeated measures (*df* = 2, 4, 8, 92) performed on data reported in Figure 3.

Parameter	F values		
	Line	Test	Line × Test
ML	3.570*	19.15****	2.394*
IL	5.000*	27.04****	2.279*
EL	4.683*	24.87****	0.3438
MF	22.56****	2.376	0.2151
IF	1.487	3.970**	0.8752
EF	8.002**	12.02****	0.9428
PEI	4.394*	4.962**	1.341
III	1.560	7.871**	0.7993
IR	11.73***	9.781****	0.7833
TCR	2.013	5.492*	0.9628
TIR	3.510*	1.115	1.790

*P < 0.05; **P < 0.01; ***P < 0.001; ****P < 0.0001.

### Basal Concentrations of Extracellular Dopamine and Glutamic Acid in Acb Shell Dialysates From DAT KO, HET and WT Rats

Under the used experimental conditions, the amounts of dopamine and glutamic acid in the dialysate obtained from DAT KO (*N* = 8), HET (*N* = 10) and WT (*N* = 8) rats that underwent five copulatory tests, and with the microdialysis probe correctly implanted in the Acb shell, were ≅54.7, 15.2 and 6.1 pg of dopamine in 20 μl of dialysate, respectively, and 0.44, 0.51 and 0.59 ng of glutamic acid in 5 μl of dialysate, respectively. These values correspond to a concentration of ≅18.70, 5.30 and 2.00 nM for extracellular dopamine, respectively, and of ≅0.60, 0.70 and 0.80 μM for extracellular glutamic acid, respectively, in DAT KO, HET and WT rats (Table 2). The above values were obtained after a 2 h perfusion period to equilibrate the Ringer’s solution with the Acb shell extracellular fluid. Since the recovery of authentic dopamine and glutamic acid of the dialysis probes was approximately 20%, extracellular dopamine and glutamic acid concentrations may be estimated to be close to ≅93.50, 26.50 and 10.00 nM for dopamine and ≅3.00, 3.50 and 4.00 μM for glutamic acid in the Acb shell of DAT KO, HET and WT rats, respectively. One-way ANOVA detected significant differences in the basal values of dopamine but not of glutamic acid concentrations among the three rat lines (calculated as the mean of the dopamine/glutamic acid values of the last four dialysate aliquots for each rat before the introduction of the female rat in the small cage of the mating cage; Table 2).

**Table 2:** Basal concentrations of extracellular dopamine and glutamic acid in the dialysate from the Acb shell of WT, HET and DAT KO rats.

Parameter	Line			One-way ANOVA	
	WT (*N* = 8)	HET (*N* = 10)	DAT KO (*N* = 8)	*F*_(2,23)_	*p*
Dopamine (nM)	1.98 ± 0.18	5.27 ± 0.40	18.72 ± 3.64****^###^	19.38	>0.0001
Glutamic Acid (μM)	0.80 ± 0.12	0.71 ± 0.07	0.59 ± 0.06	1.39	0.2692

****P < 0.0001 vs. WT. ^###^P < 0.001 DAT KO vs. HET. One-way ANOVAs followed by Tukey’s pairwise comparisons. Values are means ± SEM of the means of the last four dialysate aliquots of 8/10 rats/group collected before the introduction of the female rat in the mating cage.

### The Extracellular Dopamine and Glutamic Acid Concentrations in the Acb Shell Dialysates From DAT KO, HET and WT Rats Change Differentially During Sexual Activity

As shown in Figure 4A, the presence of the inaccessible receptive female rat and subsequent copulation with her led to an increase in extracellular dopamine concentrations in the Acb shell dialysate from DAT KO, HET and WT rats, but with significant differences among the three rat lines. These differences are due mainly to the differences found in the absolute values of dopamine in the Acb dialysate of the three rat lines (dopamine in DAT KO > HET > WT rats) rather than to differences in the temporal patterns of dopamine release, as these were found very similar among the three rat lines. Accordingly, two-way ANOVA analysis of the dopamine values of the three rat lines revealed significant effects of Line, Time and a significant Line × Time interaction (see Table 3 for F values and significance level). Moreover, pairwise comparisons showed: (i) significant differences in extracellular basal dopamine values among the three rat lines as well as during the exposition to the receptive female rat (main effect “Line”; see Figure 4A for pairwise single points of significance between rat lines); and (ii) a significant increase in the dopamine release during sexual interaction with the receptive female rat at 45 min in HET, 60 min in WT and at 75 min in DAT KO rats (with peaks of 101%, 89% and 80% above basal values, at 60 min in WT and HET and 75 min in DAT KO rats, respectively; main effect “Time”). The increments in dopamine release persisted throughout the entire copulation period, decreasing slowly to values similar to the basal ones after removal of the female rat in WT rats and, to a lesser extent, in HET and DAT KO rats (see Figure 4A for single points of significance during the experiment).

**Table 3:** F values and significance levels of two-way ANOVAs for repeated measures performed on the results shown in Figures 4, 5.

Parameter	F Values			
	Line	Time	Line × Time	*df*
Dopamine	22.60****	11.98****	4.103****	2,10, 20, 230
Glutamic acid	3.479*	15.23****	1.874*	2,10, 20, 230
NCPEs	6.911**	3.338	4.507*	2,1, 2, 23
Mounts	23.83****	1.890	4.574***	2,3, 6, 23
Intromissions	1.385	32.18****	1.616	2,3, 6, 23
Ejaculations	6.369**	11.29****	0.8957	2,3, 6, 23

*P < 0.05; **P < 0.01; ***P < 0.001; ****P < 0.0001.

As shown in Figure 4B, the concentrations of glutamic acid also increased above the basal values in the Acb shell dialysate from DAT KO, HET, and WT rats when exposed to the receptive female rat. However, although the basal values of the amino acid were found similar among DAT KO, HET and WT rats, the temporal pattern of glutamic acid release was significantly different among the three rat lines. Accordingly, two-way ANOVA analysis of glutamic acid values of DAT KO, HET and WT rats revealed significant effects of Line, Time and a significant Line × Time interaction (see Table 3 for *F* values and significance level). Moreover, pairwise comparisons (main effect “Time”) revealed a first significant increase in glutamic acid concentration after the introduction of the female rat in the mating cage, but only in WT rats (80% above basal values), whereas a higher increase in WT and also in HET rats was detected during copulation, with peak values in the first 15 min for both lines (154% and 115% above basal values, in WT and HET rats, respectively). In contrast, while no increase in glutamic acid concentration was detected in DAT KO rats either when the female rat was inaccessible or in the first 30 min of copulation, an increase in the concentration of the amino acid was found in DAT KO rats only in the second half period of copulation with a peak value at 75 min (100% above basal values; see Figure 4B for single points of significance during the experiment). Pairwise differences (main effect “Line”) in glutamic acid values among the three rat lines were also detected during copulation (from 45 up to 90 min), in particular between DAT KO and WT rats and, to a lesser extent, between DAT KO and HET rats (see Figure 4B for pairwise single points of significance among rat lines).

### The Differences in Extracellular Dopamine and Glutamic Acid Concentrations in Acb Shell Dialysates From DAT KO, HET and WT Rats Occur Concomitantly With Differences in Sexual Behavior

The differences in extracellular dopamine and glutamic acid concentrations in the dialysate from the Acb shell found in DAT KO, HET and WT rats (Figure 4) occurred concomitantly with differences in the number of NCPEs recorded when the female rat was inaccessible and in several copulatory parameters (MF, IF, EF) recorded during copulation with the available female rat (Figure 5). Accordingly, point to point analyses by two-way ANOVA (see Table 3 for F values and significance level) followed by pairwise comparisons revealed significant differences among DAT KO, HET and WT rats in all the above parameters except for the IF. Briefly, pairwise comparisons (main effect “Line”) revealed that: (i) HET rats showed more NCPEs than their DAT KO counterparts and WT rats as well; (ii) HET and, to a lesser extent, DAT KO rats showed more mounts compared to WT rats; (iii) DAT KO rats showed more ejaculations (e.g., higher EF) compared to WT and, to a lesser extent, HET rats (see Figure 5 for statistical significance of single points among lines).

Moreover, one-way ANOVA analyses of the sexual parameters recorded in the first series of copulatory activity confirmed the results of the last (fourth/fifth) copulatory tests performed before the microdialysis experiment, that is: (i) a lower ML, IL and EL in DAT KO and HET rats compared to WT rats; (ii) a higher MF in HET and lower in DAT KO rats compared to WT rats; (iii) a lower IR in HET compared to both DAT KO and WT rats; and (iv) a lower IF and higher EF in DAT KO than WT and, to a lesser extent, HET rats (Table 4). Additionally, at variance from the IR (which is calculated in the first series of copulatory activity), the total IR (TIR; obtained by dividing the number of total intromissions by the sum of total mounts and total intromissions) displayed lower values in DAT KO and HET compared to WT rats. Finally, while no difference was observed in the III during the first series of copulatory activity among DAT KO, HET and WT rats, the analysis of the TCR (which considers the number of both mounts and intromissions during the whole test) detected significantly lower values (approximately half) in DAT KO and HET compared to WT rats, a finding that indicates a general higher rate of approaching behavior to the female rat during the entire experiment in these two rat lines than that of WT rats (Table 4).

**Table 4:** Non-contact penile erections (NCPEs), copulatory parameters measured in the first series of copulatory activity (ML, IL, EL, MF, IF, III, IR, and PEI) and the total number of mounts (TMF), intromissions (TIF) and ejaculations (TEF), total copulatory rate (TCR) and total intromission ratio (TIR) of DAT KO, HET and WT rats during the entire microdialysis experiment.

Behavioral parameters	Line			One-way ANOVA	
	WT (*N* = 8)	HET (*N* = 10)	DAT KO (*N* = 8)	*F*_(2,23)_	*p*
NCPEs	1.25 ± 0.53	3.20 ± 0.68*	0.50 ± 0.19^##^	6.911	0.0045
ML (1st series)	41.00 ± 14.16	11.50 ± 1.61*	13.25 ± 2.97	4.404	0.0240
IL (1st series)	59.13 ± 24.73	15.00 ± 2.32	16.00 ± 2.56	3.464	0.0484
EL (1st series)	700.5 ± 176.1	478.6 ± 44.77	302.4 ± 16.94*	3.790	0.0378
MF (1st series)	11.88 ± 1.93	22.00 ± 2.56**	5.62 ± 0.88^####^	16.83	>0.0001
IF (1st series)	10.25 ± 1.15	8.90 ± 0.69	6.25 ± 0.88^*^	4.790	0.0182
III (1st series)	78.78 ± 23.71	55.45 ± 6.01	60.85 ± 15.80	0.604	0.5552
IR (1st series)	0.477 ± 0.03	0.297 ± 0.02*	0.533 ± 0.07^##^	7.983	0.0023
PEI (1st series)	323.6 ± 41.27	306.8 ± 15.17	226.6 ± 10.93*	4.104	0.0299
TMF	42.75 ± 6.59	121.7 ± 7.11****	69.63 ± 11.30^***^	23.83	>0.0001
TIF	29.25 ± 3.91	21.20 ± 2.13	23.25 ± 4.69	1.385	0.2704
TEF	3.12 ± 0.58	3.90 ± 0.50	5.62 ± 0.32**^#^	6.369	0.0063
TCR	46.54 ± 10.32	18.44 ± 1.82**	22.93 ± 2.61*	6.682	0.0052
TIR	0.422 ± 0.05	0.150 ± 0.02***	0.260 ± 0.04*	12.31	0.0002

*P < 0.05, **P < 0.01, ***P < 0.001, ****P < 0.0001 vs. WT. ^#^*P* < 0.05, ^##^*P* < 0.01, ^####^*P* < 0.0001, DAT KO vs. HET. One-way ANOVAs followed by Tukey’s pairwise comparisons. Values are means ± SEM of 8/10 rats/group.

### DAT KO, HET and WT Rats Display Significant Differences in Genital Self-grooming During Copulation

As shown in Figure 6, during the microdialysis experiment with the available female rat, in the first series of copulatory activity DAT KO, HET and WT rats showed also genital self-grooming after mounts, intromissions and ejaculation as expected (Sachs et al., 1988). However, the percent of mounts followed by genital self-grooming was significantly higher in DAT KO than in HET and WT rats (Chi-square test, DAT KO vs. WT: *χ*^2^ = 4.503; DAT KO vs. HET: *χ*^2^ = 3.905, both *P*s < 0.05; Figure 6A), despite the lower frequency of this behavior due to the lower number of mounts displayed by DAT KO rats (Figure 6B). This did not occur after intromissions or ejaculation, when all rats always showed genital grooming (Figure 6A). DAT KO rats had also significantly longer genital self-grooming episodes after mounts, intromissions and ejaculations compared to WT and, to a lesser extent, HET rats, which displayed intermediate time values between DAT KO and WT rats (Figure 6C). Accordingly, one-way ANOVA analyses followed by pairwise comparisons confirmed significant differences in the frequency and duration of genital self-grooming episodes among the three rat lines (see Table 5 for F values and significance level).

**Table 5:** F values and significance levels of one-way ANOVAs performed on the results shown in Figures 6B,C.

Genital self-grooming parameter	One-way ANOVA	
	*F*_(2,23)_	*p*
No. of genital grooming episodes after mounts	18.50	>0.0001
No. of genital grooming episodes after intromissions	4.79	0.0182
No. of genital grooming episodes after ejaculations	5.455	0.0115
Duration of genital grooming episodes after mounts	32.92	>0.0001
Duration of genital grooming episodes after intromissions	26.67	>0.0001
Duration of genital grooming episodes after ejaculations	38.42	>0.0001

### Differences in the Expression of trkB, BDNF, Δ-FosB, Arc, Synaptophysin, Syntaxin-3, PSD-95 and PSA-NCAM in the VTA, mPFC and Acb Among DAT KO, HET and WT rats

The antibodies against trkB, BDNF, Δ-FosB, Arc, synaptophysin, syntaxin-3, PSD-95, and PSA-NCAM recognized protein bands with a relative MW of  ≅140, 13, 36, 55, 39, 33, 80 and 206 kDa, respectively (Figures 7, 8), consistent with the expected MWs (see Carta et al., 2018; Serra et al., 2018; Sanna et al., 2019 and references therein).

As shown in Figures 7, 8, significant differences were found in the expression of trkB and BDNF, and markers of neuronal activation (Δ-FosB), synaptic function (synaptophysin, syntaxin-3, PSD-95) and plasticity (Arc and PSA-NCAM) in the VTA, the mPFC and the Acb (shell and core), limbic areas relevant for the motivational aspects of sexual behavior, among the three rat lines, in particular between WT and KO rats, although tendencies were also observed in HET rats (see Table 6 for F values and significance level from one-way ANOVAs). Indeed, in the mPFC, except Arc whose levels were higher (+66.5%) and PSA-NCAM whose lower levels did not reach statistical significance, all the other markers showed lower levels in DAT KO than WT rats [with HET rats displaying a similar trend and lower levels of PSA-NCAM (−43.6%) and syntaxin-3 (−21.1%) compared to WT rats], a finding suggestive of the presence of a lower prefrontal activation, synaptic function and plasticity in DAT KO animals (−66.9%, −58.6%, −74.8%, −51.8%, −21.2%, −86.4%, for trkB, BDNF, Δ-FosB, synaptophysin, syntaxin-3, and PSD-95, respectively; 7). Moreover, in the VTA, DAT KO rats showed lower BDNF levels (−46.9%) accompanied by a higher expression of its high-affinity receptor trkB (+57.1%), lower levels of syntaxin-3 (−29.3%), and higher levels of Δ-FosB, PSD-95 and PSA-NCAM (+64.2%, +72.2%, and +72.9%, respectively) compared to WT rats, with HET rats displaying a similar trend and lower levels of BDNF (−65.7%) and syntaxin-3 (−25.1%) than WT rats (Figure 7). Conversely, lower levels of Δ-FosB (−56, 5%), synaptophysin (−60.9%), syntaxin-3 (−58.4%) and PSD-95 (−37.3%), and higher levels of Arc (+124.9%) were observed in the Acb shell of DAT KO compared to WT rats, with HET rats displaying a similar trend and lower levels of PSD-95 (−33.6%) than WT rats (Figure 8). Finally, compared to WT rats, a higher expression of synaptophysin (+84.2%), Δ-FosB (+116%) and PSA-NCAM (+98.3%) together with lower levels of Arc (−30.6%) and PSD-95 (−42.9%) was observed in the Acb core of HET rats, with DAT KO rats displaying a similar trend and lower levels of Arc (−28.4%) than WT rats (Figure 8; see Table 6 for F values and significance levels).

**Table 6:** F values and significance levels of one-way ANOVAs performed on the results shown in Figures 7, 8.

Brain area	Marker	One-way ANOVA		
		*F*	*d.f.*	*p*
mPFC	BDNF	4.209	2,21	0.0290
	Trk-B	4.293	2,21	0.0273
	Arc	3.891	2,19	0.0383
	Δ-FosB	4.599	2,19	0.0235
	Synaptophisin	4.021	2,21	0.0332
	Syntaxin-3	4.365	2,21	0.0260
	PSD-95	4.495	2,20	0.0244
	PSA-NCAM	4.469	2,19	0.0257
VTA	BDNF	9.731	2,19	0.0012
	Trk-B	3.787	2,23	0.0379
	Arc	0.9958	2,19	0.3879
	Δ-FosB	3.972	2,19	0.0362
	Synaptophisin	2.607	2,20	0.0986
	Syntaxin-3	5.290	2,19	0.0149
	PSD-95	4.872	2,22	0.0177
	PSA-NCAM	4.217	2,21	0.0289
Acb Shell	BDNF	0.4327	2,20	0.6547
	Trk-B	1.471	2,20	0.2535
	Arc	4.319	2,20	0.0276
	Δ-FosB	4.224	2,21	0.0287
	Synaptophisin	4.116	2,20	0.0318
	Syntaxin-3	3.730	2,21	0.0411
	PSD-95	4.979	2,19	0.0183
	PSA-NCAM	0.0946	2,21	0.9100
Acb Core	BDNF	0.4706	2,21	0.6311
	Trk-B	0.8223	2,21	0.4531
	Arc	5.469	2,21	0.0123
	Δ-FosB	3.719	2,21	0.0414
	Synaptophisin	3.636	2,20	0.0450
	Syntaxin-3	0.5830	2,19	0.5679
	PSD-95	4.853	2,19	0.0198
	PSA-NCAM	4.076	2,19	0.0337

## Discussion

This study shows for the first time that DAT KO rats (Leo et al., 2018b) exhibit patterns of sexual behavior different from those of their matched HET and WT counterparts when put together with a sexually receptive (ovariectomized and primed with estradiol and progesterone) female rat. The differences in the sexual patterns and associated copulatory parameters in DAT KO, HET and WT rats are more evident in the first copulatory test and tend to stabilize, but not disappear, after five copulatory tests. This study also shows that once stabilized, the patterns of sexual behavior in DAT KO, HET, and WT rats are related to differential changes among the three rat lines in the release of dopamine and glutamic acid in the shell of the Acb, and differences in the expression of Δ-FosB, synaptophysin, syntaxin-3, BDNF, trkB, PSD-95, PSA-NCAM and Arc in the medial PFC, in the VTA and the Acb shell and core.

### DAT KO, HET and WT Rats Show Different Patterns of Sexual Behavior With a Sexually Receptive Female Rat

DAT KO, HET and WT rats showed significant differences in the first copulatory test when put together with a sexually receptive female rat. Accordingly, while 60% of WT rats engaged in sexual behavior with mounts and intromissions, 75% of HET rats and 90% of DAT KO rats did so with 90% of DAT KO and 60% of HET rats achieving ejaculation already in the first test against the 50% of the WT counterpart. The differences between DAT KO and HET rats disappeared in the second test, while those between DAT KO and HET rats and WT rats disappeared in the third test when, irrespective of the belonging rat line, all rats (e.g., 100%) engaged in sexual activity and reached ejaculation. Although the Chi-square (*χ*^2^) test failed to reveal any statistical significance on these differences due to the relatively low number of animals used, these findings seem to indicate a faster approach directed to and/or a higher ability to sexually interact stably and efficiently with the female rat in DAT KO rats compared to HET rats and, even more, to WT rats. This occurred together with the typical significant improvements in sexual parameters recorded during the first series of copulatory activity during the five tests in DAT KO, HET and WT rats, e.g., a decrease of the ML, IL, and EL, an increase of the EF, and an improvement of the IR. However, this general trend apart, significant differences occurred among DAT KO, HET, and WT rats, with DAT KO rats constantly displaying higher EF and IR values, and lower MF and shorter ML, IL and EL values already during the first test and in the subsequent ones compared to WT and also to HET rats, which displayed values similar to those of WT rats in the majority of parameters during the first and following tests, except for minor improvements in IR and higher MF values among all the five tests, compared to WT rats. This last result together with the differences in the IF values (lower for DAT KO rats, similar between HET and WT rats) is responsible for the significantly lower IR values of HET compared to DAT KO and WT rats in the last test. HET and DAT KO rats showed also a lower TIR [(total intromission ratio) = TIF/(TIM + TIF)] and a significantly shorter (approximately half) TCR (total copulatory rate). Together with a higher level of sexual motivation and sexual activity in DAT KO rats inferred the first by the lower ML and IL, and the second by the higher EF and IR shown in the first and following tests as well, compared to WT and HET rats, these results account for faster acquisition of the ability to sexually interact stably and efficiently with a receptive female rat, of DAT KO rats compared to HET and mainly WT rats.

In line with the results of this study, sexual patterns similar to those observed in DAT KO rats are seen in rats treated with drugs that block DAT function, mainly cocaine (reviewed in Frohmader et al., 2010; Pfaus et al., 2010). Accordingly, acute cocaine treatment facilitates penile erection, reduces the number of intromissions, increases the number of mounts and shortens the ejaculation latency (that sensitizes with chronic administration of cocaine over time leading to higher ejaculation frequency). These data suggest that cocaine facilitates ejaculation when given acutely and even more after chronic administration and, although acute cocaine increases ML and IL, tolerance develops to these effects. Moreover, the lower number of intromissions together with the higher number of mounts account for a lower intromission rate in cocaine-treated rats. Although cocaine inhibits not only DAT but also noradrenaline (NET; see Tatsumi et al., 1997) and serotonin (SERT) transporters (see Rudnick and Sandtner, 2019), together, the findings of this study and those of cocaine-treated rats, apparently confirm that the absence (as in DAT KO rats) or the inactivation of DAT function (as in cocaine-treated rats) in areas involved in sexual behavior, leads to alterations of sexual behavior by impacting, in particular, the latency and the frequency of ejaculation (which become the first shorter and the latter higher, respectively) and the intromission rate [i.e., number of intromissions/(number of mounts + number of intromissions)], due to changes in the number of mounts and intromissions, the first being increased and the latter decreased in both DAT KO and in cocaine-treated rats.

According to previous reports (Sachs et al., 1988), this study also shows that mounts, intromissions and ejaculations were followed by bouts of genital grooming in DAT KO, HET and WT rats. These bouts were seen more frequently in DAT KO compared to HET and WT rats after mounts, but not after intromissions and ejaculation, and lasted longer in DAT KO rats and, to a lesser extent in HET rats, when compared to WT rats. The reason why a higher percent of mounts are followed by genital self-grooming episodes in DAT KO rats as well as why DAT KO rats are those that show the longest duration of genital self-grooming after mounts, intromissions and ejaculation among the three rat lines, is unknown at the moment. However, since excessive grooming has been considered a putative symptom of compulsive behavior (Berridge et al., 2005; Feusner et al., 2009) when associated to conditions of elevated dopamine or higher serotonin activity (Bagdy et al., 1992; Berridge et al., 2005; Taylor et al., 2010), given the tendency of DAT KO rats to show compulsive behavioral traits (Adinolfi et al., 2018, 2019; Cinque et al., 2018), the long-lasting genital self-grooming found in DAT KO rats after mounts, intromissions and ejaculation, may be interpreted as a further compulsive trait.

### Extracellular Dopamine and Glutamic Acid Concentrations in the Dialysate Obtained From the Acb Shell of DAT KO, HET and WT Rats With Stable Levels of Sexual Behavior During Sexual Activity With a Receptive Female Rat

This study also shows that DAT KO, HET, and WT rats, which underwent five copulatory tests and show different patterns of sexual behavior and associated values of copulatory parameters (see above), have different basal concentrations of extracellular dopamine in the Acb shell dialysate (e.g., when no sexual receptive female rat was present, extracellular dopamine concentration in DAT KO rats was 9-fold and in HET rats 2–3 fold higher, respectively, than that of WT rats). This finding resembles the higher levels of extracellular dopamine found in the dialysate obtained from the dorsal striatum of DAT KO rats compared to those of HET and WT rats (Leo et al., 2018b). Irrespective of the different basal levels of extracellular dopamine in the Acb dialysate among DAT KO, HET, and WT rats, dopamine levels increased in the dialysate of the Acb shell of DAT KO, HET and WT rats when a sexually receptive female rat was introduced in the mating cage, either when the female rat was inaccessible (e.g., the male can see, hear and smell, but not interact with her) or when copulation was allowed, as expected (see Fiorino et al., 1997; Sanna et al., 2015b). Most importantly, the patterns of release in response to the presence of the inaccessible female rat first, and then to the direct interaction with her, were very similar among the three lines, with peak increases of 22%, 46%, and 59%, respectively, at 15 min with the inaccessible female rat, and of 80%, 89%, and 101%, respectively, after 30–45 min of direct sexual interaction with the receptive female rat, in DAT KO, HET and WT rats. These increases lasted, although reduced, for the entire experiment, and decreased after the removal of the female rat from the mating cage.

At variance from dopamine, this study failed to reveal significant differences in the basal levels of extracellular glutamic acid in the same dialysate obtained from the Acb shell used for dopamine measurement in DAT KO, HET and WT rats. Nevertheless, the patterns of release in response to the presence of the inaccessible sexually receptive female rat first, and then to the direct interaction with her, were also similar between WT and HET rats, but with significant differences in DAT KO rats. Accordingly, glutamic acid increased of 32.8% and 80.4%, respectively, at 30 min with the inaccessible female rat, and of 114.9% and 153.5%, respectively, after 15 min of direct sexual interaction with the receptive female rat, respectively, in HET and WT rats. These increases also lasted, although substantially reduced, for the entire experiment and decreased after the removal of the female rat from the mating cage. Surprisingly, at variance from HET and WT rats, no increase in glutamic acid levels was found either during the presence of the inaccessible female rat and up to 45 min of copulation in DAT KO rats, after which a peak increase of 99.3% was observed that lasted, although reduced, until the end of the copulatory test and removal of the female rat from the mating cage.

Perhaps more importantly, the increases in extracellular dopamine and glutamic acid levels, which occurred in the Acb shell dialysate when the sexually receptive female rat was introduced in the mating cage, were parallel to differential changes in the values of the copulatory parameters (i.e., MF, IF and EF) similar to those found after stabilization of copulatory behavior in the forth/fifth copulatory test, among DAT KO, HET, and WT rats. Indeed, also in this experiment, HET rats showed higher MF than DAT KO and WT rats mainly in the first 30 min of copulation, and a lower EF (similar to that of WT rats) than DAT KO rats, despite a similar IF among the three rat lines. Likewise, HET and DAT KO rats (which displayed more mounts than WT rats) showed also a lower TIR [total intromission ratio = TIF/(TIM + TIF)] and a significantly shorter (approximately half) total copulatory rate (TCR; i.e., they displayed a higher number of approaches to the female rat per time unit) compared to WT rats. As expected, HET and WT rats showed also NCPEs, with HET rats showing more NCPEs than WT rats, but surprisingly these penile erections did not occur in DAT KO rats.

The above differences in the pattern of release of dopamine and glutamic acid in the Acb shell and NCPEs and copulatory parameters among DAT KO, HET and WT rats, deserve some comment. First, the higher basal levels of dopamine found in the Acb shell dialysate from DAT KO rats compared to those of HET and WT rats raise the possibility that a higher basal dopaminergic tone exists in the Acb shell of DAT KO rats and that this enhanced tone may be responsible, at least in part, for the higher sexual motivation and for the higher levels of sexual activity found in these animals compared to their HET and WT counterparts (see above). Indeed, a higher dopaminergic tone may contribute to making these animals more prone to interact with the receptive female rat, thereby facilitating the first interaction with her and, as a consequence, accounting for faster acquisition of the ability to stably sexually interact with the receptive female rat than their HET and WT counterparts. In line with the above hypothesis, we have recently reported that a different mesocorticolimbic dopaminergic tone correlates with differences in several aspects of sexual behavior, from the acquisition of sexual experience to sexual motivation and performance, in RHA and RLA rats (see the “Introduction” section; Sanna et al., 2015b, 2017b). RHA rats exhibit higher levels of sexual motivation and better performances than RLA rats (Sanna et al., 2014a) both in naïve and sexually experienced conditions, and these sex differences are secondary to the more robust functional dopaminergic tone occurring in RHA compared to RLA rats (Sanna et al., 2013, 2014b, 2015b, 2017b).

However, the explanation given above that a higher dopaminergic tone of the mesolimbic system is responsible for a higher level of sexual motivation and sexual activity in DAT KO rats compared to HET and WT rats is complicated to some extent by a few findings. First, at variance from DAT KO, HET and WT rats, the higher dopamine release of RHA vs. RLA rats occurs without any difference in the basal levels of extracellular dopamine in the Acb and mPFC dialysate of these two rat lines (e.g., basal extracellular dopamine levels are similar in the Acb and mPFC dialysate from RHA and RLA rats; Giorgi et al., 2007; Sanna et al., 2015b, 2017b). Second, there is no correlation between the dopamine concentrations found in the Acb dialysate from DAT KO, HET and WT rats and the number of concomitant NCPEs shown by the three lines when exposed to the inaccessible female rat. NCPEs are pheromone-induced penile erections which a male rat displays when put in the presence of an inaccessible receptive female rat and are considered an index of sexual arousal (Sachs et al., 1994). NCPEs usually occur concomitantly with an increase in extracellular dopamine levels in areas relevant for a role of dopamine in sexual behavior, e.g., the paraventricular nucleus of the hypothalamus (Melis et al., 2003), the Acb shell (Sanna et al., 2009, 2015b) and the mPFC (Sanna et al., 2017b). This led to suggest that male rats that display more NCPEs, display also a higher dopamine release in these areas and the Acb in particular, compared to rats displaying less NCPEs (Sanna et al., 2009, 2015b). In line with the above hypothesis, DAT KO rats, which show the lower dopamine increases (22% above basal values) among the three rat lines, fail to display NCPEs; however, against the above hypothesis, HET and WT rats, which show dopamine peaks of 46% and 59% above basal levels, respectively, showed a mean of 2 and 0.8 NCPEs, respectively, that is HET rats showed 2-fold more NCPEs than WT rats, irrespective of the fact that WT rats had the highest dopamine release and should be then expected to show the highest number of NCPEs. Further complications also arise from the different basal levels of extracellular dopamine in the Acb dialysate from DAT KO, HET and WT rats. Indeed, since basal dopamine levels are about 3/4-fold higher in DAT KO rats than those in HET rats and 9-fold higher than those in WT rats, one should also expect that DAT KO rats would have shown more NCPEs than HET rats, and HET rats more NCPEs than WT rats. In contrast to this hypothesis and as discussed above, DAT KO rats failed to show NCPEs and HET rats showed more NCPEs than WT rats. Although further work is necessary to clarify whether NCPEs appearance is secondary to the increase in dopamine output more than to the absolute amount of extracellular dopamine in the extracellular fluid, the finding that DAT KO rats do not show NCPEs together with the finding that these rats show the lowest dopamine increases among the three rat lines, suggest that the increase in dopamine release rather than the basal levels of the neurotransmitter in the extracellular fluid may play a major role in mediating this sexual response. Finally, DAT KO and HET rats (both having basal dopamine levels higher, and displaying more mounts than WT rats, see Figure 5), compared to WT rats, show a lower TIR [total intromission ratio = TIF/(TMF + TIF)] that occurs despite what seen in ML, IL, EL and EF (the first three decrease and the latter increases more in DAT KO and HET than WT rats). Indeed, this finding suggests that the higher dopaminergic tone is related to the total number of approaches (i.e., the sum of mounts and intromissions during the copulatory test) performed by HET and DAT KO rats compared to WT rats rather than to their level of performance, usually measured by the IR (intromission ratio). Accordingly, both DAT KO and HET rats display not only a reduced TIR but also a significantly shorter (approximately half) total copulatory rate (TCR; they display a higher number of approaches to the female rat per time unit) compared to WT rats.

As to the increase in Acb glutamic acid output found in DAT KO, HET and WT rats, these findings provide evidence for a role of this excitatory amino acid in sexual behavior in the Acb shell. To our knowledge, this study is the first to show that: (i) extracellular glutamic acid levels increase in the Acb shell dialysate during sexual behavior; (ii) this increase occurs concomitantly to an increase in extracellular dopamine and (iii) it is associated to differences in the pattern of sexual behavior in DAT KO, HET and WT rats. So far, only a few other studies provided evidence for a role of Acb glutamic acid in sexual behavior. Among these, one shows that the acquisition of sexual experience causes long-term alterations in glutamate receptor expression and function (i.e., a decrease in AMPA/NMDA receptors ratio) and that changes in the NMDA (NR1 subunit) and AMPA (GluA1 and GluA2 subunits) receptors are differentially associated to experience acquisition, reward and abstinence, pointing out to a complex fine-tuning (i.e., changes in receptor subunits trafficking) at the synaptic level of the neuroplastic processes occurring during the acquisition of sexual experience in male rats (Pitchers et al., 2012). Another one shows that in female Syrian hamsters a glutamatergic projection from the medial PFC to the Acb is activated during sexual behavior and that silencing these mPFC glutamatergic afferents (with DREADD techniques) to the Acb prevents C-Fos expression in the Acb due to sexual interaction, although this silencing did not affect female sexual behavior expression, thus excluding a role in sexual performance and pointing out on a possible role in the motivational (i.e., anticipatory and rewarding) aspects of sexual activity (Moore et al., 2019). The other available studies support a facilitatory role of glutamic acid in sexual behavior in brain areas such as the PVN and the medial preoptic area of male rats. Accordingly, extracellular glutamic acid (and aspartic acid), was found to be increased in the paraventricular dialysate during the exposition to an inaccessible receptive female rat and even more during copulation (Melis et al., 2004; here the excitatory amino acid facilitates male sexual behavior by activating central oxytocinergic neurotransmission, see Melis and Argiolas, 2011; Argiolas and Melis, 2013) and in the medial preoptic area dialysate, where the excitatory amino acid increased maximally at ejaculation (Dominguez et al., 2006). However, at variance from Acb extracellular dopamine, whose levels increase during sexual behavior and for which an increased tone has been already involved in the different patterns of sexual behavior of several rat lines (see Fiorino et al., 1997; Sanna et al., 2015b, 2017b; Melis et al., 2019), allowing us to suggest that the different sexual patterns found in DAT KO, HET and WT rats may be related to the differential changes in the Acb dopamine output (see above), further studies are necessary to clarify if and how the different patterns of release of glutamic acid found in the Acb of DAT KO, HET, and WT rats are related to the differences in sexual patterns and copulatory parameters of these three rat lines. Nevertheless, it is reasonable to assume that some, if not all, of the above differences in sexual behavior among DAT KO, HET and WT rats may be mediated, at least in part, by an altered interaction between Acb dopamine and glutamic acid. Infact, it is well accepted that: (i) the function of mesolimbic and mesocortical dopaminergic neurons, which play a key role in motivated behavior (Goto and Grace, 2005) and have their cell bodies mainly in the VTA, is finely modulated by the activity of glutamic acid neurons, which originate, although not exclusively, from the PFC and project to the Acb and to the VTA (Carlsson et al., 2001; Carlezon and Thomas, 2009; Beloate and Coolen, 2017; Bamford et al., 2018); (ii) an intact glutamatergic function in the PFC is required for cortical control over the activity of the dopaminergic mesolimbic system to modulate, together with dopamine release, the activity of the GABAergic medium spiny neurons at the level of the Acb and motivated behavior (Carlsson et al., 2001; Kalivas, 2009); and (iii) dopamine and glutamic acid, together with the neuropeptide oxytocin, are key neurotransmitters in the functioning of a complex brain circuit, interconnecting hypothalamic, limbic and cortical areas, involved in both the motivational and performance aspects of male rat sexual behavior (Melis et al., 2007, 2009, 2010; Succu et al., 2007, 2008, 2011; Melis and Argiolas, 2011; Sanna et al., 2017a; Bratzu et al., 2019).

### Differences in the Expression of trkB, BDNF, Δ-FosB, Arc, Synaptophysin, Syntaxin-3, PSD-95 and PSA-NCAM in the VTA, mPFC and Acb of DAT KO, HET and WT Rats Are Related to the Differences in the Patterns of Sexual Activity Among the Three Rat Lines

This study also shows for the first time that the relative protein levels of markers of neurotropism (BDNF, trkB), neuronal activation (Δ-FosB), synaptic function (synaptophysin, syntaxin-3, and PSD-95) and plasticity (Arc and PSA-NCAM; Carta et al., 2018; Serra et al., 2018; Sanna et al., 2019 and references therein) are differentially expressed in the VTA, mPFC and Acb of DAT KO, HET and WT rats, which underwent five copulatory tests and a final session of sexual activity performed during intracerebral microdialysis with a sexually receptive female rat. The most significant differences were detected mainly in the mPFC, where a lower expression of almost all the markers investigated, except for Arc (whose expression was higher) and PSA-NCAM (whose lower levels did not reach statistical significance), was found in DAT KO compared to WT rats, with HET rats usually showing intermediate changes between the other two. Differences in the expression of the majority of the above markers were also detected in the VTA and the Acb shell and core; however, only in a few cases, these differences were in the same direction of those found in the mPFC among the three rat lines. Thus, comparing DAT KO rats to WT ones, BDNF was lower and trkB higher in the VTA, with no differences in the Acb core and shell; Δ-FosB was higher in the VTA, but lower in the Acb shell; synaptophysin was lower in the Acb shell; syntaxin-3 was lower in the VTA and the Acb shell; PSD-95 was higher in the VTA, and lower in the Acb shell; PSA-NCAM was higher in the VTA but did not differ in the Acb shell; Arc, which was higher in the mPFC and Acb shell, was lower in the Acb core and did not differ in the VTA.

Interestingly, in line with our previous observations in the limbic system of Roman rats (Sanna et al., 2015b, 2017b, 2019 and references therein), the differences in the markers’ expression in the VTA, mPFC and Acb among DAT KO, HET and WT rats parallel the different sexual patterns and the differential changes found during sexual activity among the three rat lines in the activity of the mesolimbic dopamine and frontocortical glutamic acid neurons, which interconnect these brain areas, with the higher difference in extracellular dopamine and glutamic acid levels in the Acb shell dialysate found between DAT KO and WT rats. Since DAT KO rats lack one of the most important regulators of dopamine function at synaptic level (Kuhar et al., 1990), and thus bear permanent alterations in the processes of neuronal plasticity and tropism driven by it (Fosnaugh et al., 1995; Fasano et al., 2013; Pitchers et al., 2013; Collo et al., 2014), and DAT KO rats are also those that show the majority of differences compared to WT rats in the expression of the markers investigated in the VTA, mPFC and Acb, it is then likely that (i) these differences are due to the absence of DAT, which leads to a reduced/altered neuronal activation and synaptic function and plasticity in the above brain areas of DAT KO compared to HET and WT rats, and (ii) a causal relationship may exist among the distinct tissue expression profiles of these markers in DAT KO, HET and WT rats and the differences in behavioral sexual patterns, copulatory parameters, and dopamine and glutamic acid release in the Acb shell. Accordingly, and in line with previous studies in mice and rats with the DAT KO genotype (Fumagalli et al., 2003; Yao et al., 2004; Leo et al., 2018b), we found a lower expression of most of the markers investigated mainly in the mPFC of DAT KO vs. WT rats. These differences would cause in DAT KO rats a weakened/altered prefrontal control over the mesolimbic systems dedicated to the translation of motivational drives into goal-directed behaviors (Goto and Grace, 2005), and lead in turn, to minor flexibility in response to changes in environmental demands such as those by sexual behavior. Thus, as already discussed above (see “Discussion” section “Extracellular Dopamine and Glutamic Acid Concentrations in the Dialysate Obtained From the Acb shell of DAT KO, HET and WT Rats With Stable Levels of Sexual Behavior During Sexual Activity With a Receptive Female Rat”), the different sexual responses of DAT KO vs. WT rats may be secondary to the altered activity of both prefrontal glutamatergic neurons and mesolimbic dopaminergic neurons, which may determine, at least in part, the different markers’ expression found in the mPFC, VTA, Acb shell and core among the three rat lines.

Mechanisms involved apart, the significance of the differences among markers’ expression across the three rat lines is also unknown, as only a few studies have investigated their expression concerning sexual behavior, mainly focusing on BDNF, trkB, Δ-FosB, and Arc (Pitchers et al., 2010b, 2013; Sanna et al., 2019; Turner et al., 2019).

As discussed above, in line with a weakened prefrontal control of DAT KO rats vs. their HET and WT counterparts, our results are in agreement with lower levels of BDNF and trkB in the mPFC reported in DAT KO mice (Fumagalli et al., 2003; Yao et al., 2004) and rats (Leo et al., 2018b). They further show that a decrease in BDNF expression also occurred in the VTA of DAT KO rats, though accompanied by an increase rather than a decrease in trkB expression when compared to HET and WT lines, while revealed no significant differences in both BDNF and trkB levels in the Acb shell and core among the three rat lines. If this decrease in the expression of both BDNF and trkB in the mPFC of DAT KO rats is related to the fact that these rats are those showing the higher basal levels of extracellular dopamine, or the lowest dopamine release during sexual activity, in the Acb shell is unknown. No help in this circumstance is provided by our previous findings on the expression of BDNF and trkB in RHA and RLA rats (Sanna et al., 2019). Indeed similar levels of BDNF were found in the mPFC, VTA and Acb, shell and core, of RHA and RLA rats after five copulatory tests, even though RHA rats have a higher dopaminergic tone in the mPFC and the Acb shell than RLA rats. Likewise, no significant differences were detected in trkB levels between RHA and RLA rats in the above brain areas except for the mPFC, in which higher levels of trkB levels were found in RLA vs. RHA rats (Sanna et al., 2019).

As to Δ-FosB, a truncated form of C-Fos, its expression pattern higher in the VTA and Acb core but lower in the mPFC and in the Acb shell of DAT KO rats, which occurred even though among the three lines the DAT KO one had the highest levels of extracellular dopamine in the Acb shell, is certainly surprising. Indeed, this marker of neuronal activation is believed to play a key role in mediating the rewarding properties of sexual behavior and in the acquisition of sexual experience, as it has been found robustly increased in the Acb shell and the mPFC, either in response to the rewarding properties of sexual activity (Pitchers et al., 2010b) or in male rats with a higher dopaminergic tone in the Acb shell and mPFC (e.g., RHA compared to RLA rats; Sanna et al., 2019). Thus, in line with the above results, DAT KO rats should be those with the highest levels of Δ-FosB in the Acb shell, as in the striatum of DAT KO mice, where basal levels of extracellular dopamine were reported to be 5-fold higher than their WT counterparts (Cyr et al., 2003). One explanation for this discrepancy may be that DAT KO rats are also those that show the lowest increase in dopamine release in the Acb shell during sexual activity when compared to WT rats. Together, the lower Δ-FosB expression and dopamine increases suggest that it is the dopamine output itself rather than the absolute dopamine amount in the synaptic cleft to drive the increase in Δ-FosB expression. However, it is also possible that Δ-FosB expression in DAT KO rats is lower because these rats are also those that show the lowest levels of glutamic acid release in the Acb shell during sexual activity and this could have interfered with its dopamine-induced activation (Beloate and Coolen, 2017). Interestingly, in this regard, inhibition of the mPFC glutamatergic input to the Acb has been found to prevent C-Fos expression in this area during sexual activity in the female hamster (Moore et al., 2019). The above hypotheses may also be not mutually exclusive.

As to the expression of Arc, a marker of neural activity and structural plasticity (Bramham et al., 2010; Korb and Finkbeiner, 2011), this was found to be higher in the mPFC and Acb shell, but lower in the Acb core of DAT KO rats compared to WT rats. These findings are in contrast with the minor difference found in RHA and RLA rats after five copulatory tests, that is after reaching a stable level of sexual activity as done for DAT KO, HET and WT rats used in this study. However, they resemble the higher increases in RHA vs. RLA rats, after the first copulatory test, a finding that is in line with the fact that Arc protein is synthesized and then transported at postsynaptic sites where it accumulates to be used when appropriate stimuli linked to new relevant experiences, as it may be considered the first exposition to a sexually receptive female and copulation, occur. Further studies are necessary to explain why, at variance from the Roman lines, DAT KO, HET and WT rats that have reached stable levels of sexual activity have different levels in Arc expression in the mPFC and Acb shell. Since the Arc protein expression pattern is modulated by changes in AMPA receptor-mediated glutamic acid transmission (Bramham et al., 2010; Korb and Finkbeiner, 2011; but see also Pitchers et al., 2012), and reduction in Arc levels as in Arc KO mice leads to changes in both AMPA receptor trafficking (Chowdhury et al., 2006) and mesocortical/mesolimbic dopamine activity (which are the first inhibited and the second activated by Arc gene silencing; Managò et al., 2016; Managò and Papaleo, 2017), it can be assumed that the differences in both glutamic acid and dopamine release, as detected in the Acb shell of DAT KO, HET, and WT rats, might be involved in the brain regional differences of Arc expression levels among the three DAT rat lines as well as between the DAT KO (present data) and the Roman lines (Sanna et al., 2019).

A lower glutamic acid release may be also involved in the lower expression of PSD-95 found in the mPFC and the Acb shell in DAT KO compared to WT rats. Accordingly, this protein, which plays a role in synaptic plasticity and the dendritic spine remodeling (together with BDNF; El-Husseini et al., 2000; de Bartolomeis et al., 2014), has been identified as a regulator of dopamine-mediated synaptic and behavioral plasticity and its expression is markedly reduced in the presence of a decreased activity of frontocortical glutamatergic projections to the Acb in DAT KO mice (Yao et al., 2004; Efimova et al., 2016). Likewise, the higher expression of PSA-NCAM in the VTA and Acb core and its lower expression in the mPFC of DAT KO and HET rats may also be related to the altered dopamine/glutamic acid function of these animals when compared to WT rats. Accordingly, the enzymatic removal of PSA (polysialic acid) from PSA-NCAM was found able to decrease spine density and to reduce the expression of vesicular glutamate transporter-1 in apical dendrites of mPFC pyramidal neurons without affecting their inhibitory innervation in male rats (Castillo-Gómez et al., 2016).

Although additional studies are necessary to ascertain if and how the different levels of expression of the above markers across the VTA, mPFC, Acb shell and core are directly related to DAT gene silencing as well as to the differential changes found between DAT KO and WT rats in the dopamine and/or glutamic acid output in the Acb shell and to the different patterns of sexual behavior, this study provides strong evidence that DAT KO and, to a lesser extent, HET rats, compared to WT rats, which have reached stable levels of sexual activity, show differences at several levels in the plastic processes occurring in these brain areas. Since sexual behavior, although highly stereotyped and instinct-guided, is also influenced by learning as other rewarding behaviors (see Pfaus et al., 2012), and learning activates plastic processes which, in turn, cause a broad array of functional and structural changes that involve neurotransmitters and their receptors, immediate early genes and their products and other molecules controlling neuronal tropism and synaptic plasticity as well (Leuner et al., 2010; Pitchers et al., 2010a, 2012; Sanna et al., 2015b, 2017b, 2019; Turner et al., 2019), these changes may have a pervasive impact on sexual behavior and, more in general, on the motivated behavior of these animals.

## Conclusions

In conclusion, this study shows that DAT KO rats and, to a lesser extent, HET rats, present differences in the motivational and consummatory aspects of sexual behavior when compared to WT matched controls. DAT KO rats needed a lower number of copulatory tests to reach a stable level of sexual activity with a sexually receptive female rat and presented higher levels of sexual activity as indicated by the changes in copulatory parameters across the copulatory tests, mainly shorter latencies to mount, intromit and ejaculate and higher ejaculation frequencies when compared to WT rats. DAT KO rats showed also a lower number of NCPEs when put in the presence of an inaccessible receptive female rat. These sexual differences among DAT KO, HET and WT rats that have reached a stable level of copulatory activity occurred concomitantly to significant differences in the basal levels of extracellular dopamine in the dialysate obtained by intracerebral microdialysis from the Acb shell (DAT KO rats show dopamine levels much higher than HET and, even more, WT rats), but not to basal levels of extracellular glutamic acid (which were similar among the three rat lines), and to significant differences in extracellular dopamine and glutamic acid release during sexual activity. Indeed, although both dopamine and glutamic acid concentrations increased above basal values in WT more than in DAT KO rats during sexual activity, with dopamine concentration increased during the entire period of sexual activity in all the three rat lines, glutamic acid increased in DAT KO rats only in the last period of sexual activity, at variance from WT and HET rats, in which it increased significantly already in the first period of sexual activity. It is reasonable to assume that these differential changes in dopamine and glutamic acid outputs are involved in the differences in sexual behavior among the three rat lines, since dopamine and glutamic acid, together with the neuropeptide oxytocin, are key neurotransmitters of a complex brain circuit that interconnects hypothalamic, limbic and cortical areas involved in both the motivational and performance aspects of the male rat sexual behavior (Melis and Argiolas, 2011; Argiolas and Melis, 2013; Sanna et al., 2017a; Bratzu et al., 2019; Melis et al., 2019). Finally, differences were also detected among DAT KO, HET and WT rats in the expression of BDNF and its trkB receptor, markers of neural activation (Δ-FosB) and plasticity (Arc and PSA-NCAM) and synaptic structural proteins (synaptophysin, syntaxin-3, PSD-95) in the VTA, mPFC, and Acb (shell and core), which are all brain areas relevant for sexual motivation and sexual performance. The expression of the majority of these markers, excluding Arc, is decreased in the mPFC, in line with the assumption of a reduced function of this brain area in DAT KO rats (Leo et al., 2018b).

Taken together, the results of this study confirm a key role of dopamine in both the motivational and performance aspects of sexual behavior, and confirm that conditions characterized by permanently high levels of dopamine (i.e., “hyperdopaminergia”), like those due to DAT gene silencing or to chronic treatment with psychostimulants (e.g., cocaine and amphetamines) in laboratory animals, may be useful for modeling the behavioral, neurochemical and molecular features not only of psychopathological conditions such as psychotic states and attention-deficit/hyperactivity disorders (ADHD) but also to characterize the neural substrates underlying conditions such as hypersexuality and altered sexual behavior.

## Data Availability Statement

The datasets generated for this study are available on request to the corresponding author.

## Ethics Statement

The animal study and the experiments were reviewed and approved by the Ethical Committee for Animal Experimentation of the University of Cagliari.

## Author Contributions

FS, AA, and MM designed the study. FS and JB designed, performed and analyzed the data from sexual behavior and microdialysis experiments. MS, MQ, and MB performed the Western Blot experiments and analyzed Western Blot data. RG, DL, and SE bred and selected DAT KO, HET and WT rats. FS, AA, MM, and MQ supervised the study. FS, AA, MM, and MQ wrote the manuscript. All authors discussed the results and commented on the manuscript.

## Conflict of Interest

The authors declare that the research was conducted in the absence of any commercial or financial relationships that could be construed as a potential conflict of interest.

## Footnotes

^1^https://www.licor.com/bio/image-studio-lite/download
